# Alterations in the activity and sleep of *Drosophila melanogaster* under simulated microgravity

**DOI:** 10.1038/s41526-021-00157-5

**Published:** 2021-07-22

**Authors:** Hongying Zhang, Yahong Wang, Ziyan Zhang, Lu Zhang, Chao Tang, Boqun Sun, Zhihao Jiang, Bo Ding, Peng Cai

**Affiliations:** 1 Key Lab of Urban Environment and Health, Institute of Urban Environment, Chinese Academy of Sciences, Xiamen, China; 2 University of Chinese Academy of Sciences, Beijing, China; 3 Xiamen Key Laboratory of Physical Environment, Xiamen, China; 4 Shanghai Institute of Nutrition and Health, Chinese Academy of Sciences, Shanghai, China

**Keywords:** Risk factors, Molecular biology, Fatigue, Environmental sciences

## Abstract

This study aimed to investigate alterations in the activity and sleep of *Drosophila melanogaster* under simulated microgravity, which was implemented through the random positioning machine, while different light conditions (normal photoperiod and constant dark) were set. Fruit flies of different strains and sexes were treated for 3 days, and activity and sleep were monitored using the Drosophila Activity Monitoring System. After 3 days of treatment, fruit flies were sampled to detect the relative expression levels of the major clock genes and some neurotransmitter-related genes. The results showed that for the normal photoperiod (LD) condition, the activity increased and sleep decreased under simulated microgravity, while for the constant dark (DD) condition, the activity and sleep rhythms appeared disordered and the activity increased, thus decreasing the likelihood of waking up during the day. Light conditions, strains, and sexes, individually or in combination, had impacts on the simulated microgravity effects on behaviors. The clock genes and neurotransmitter-related genes had different degrees of response among sexes and strains, although the overall changes were slight. The results indicated that the normal photoperiod could ease the effects of simulated microgravity on fruit flies’ activity and sleep and possible unidentified pathways involved in the regulatory mechanism need further exploration. This study is expected to provide ideas and references for studying the effects of microgravity on space life science.

## Introduction

Gravity is a relatively constant physical factor on Earth, and all living organisms are affected and well adapted to the gravitational field^1,2^. Since the beginning of space flight, scientists have been investigating the effects of microgravity on living organisms because these effects must be considered in space flight programs for the exploration of space.

Behavior is one of the relatively plastic properties of living organisms, especially in complex multicellular systems; therefore, changes in behavior are one of the relatively common characteristics of changes in environmental conditions, such as changes in gravity^2–4^. Sleep is an important behavior, and sleep alterations were recognized shortly after manned space flight began. Several studies have shown that astronauts have sleep disturbances during orbit, such as short sleep times, poor sleep quality and disruption of sleep^5–11^. Astronaut log data and actigraphy documents showed that the mean estimated sleep duration during orbit was reduced to ~6 h/night, which is 1.5–2 h less than the recommended 8 h and significantly less than the sleep time at ~3 months prior to and 1 week immediately after flight^5,6,9,12^. In addition, some studies have shown that sleep time fluctuates greatly each night and sleep characteristics change, which might further increase the likelihood of waking neurobehavioral performance deficits^7,10,13,14^. The likelihood and consequence of performance errors due to sleep disturbances are dangerous^6,13,15^. In addition, the use of sleep medications, including sleep and wake-promoting medications, is also widely recorded among astronauts^9,16^ and occurs at a rate 10–20 times higher than that of the general population^17^. However, some research suggested that no significant alterations in sleep parameters during orbit occurred sometimes after administering sleep medications^7,8^. In addition, the efficacy, stability, safety, and effectiveness of these medications in space and potential interactions with other non-sleep medications need vigilance and further investigation^6–9,14,16,18–20^. Nevertheless, a growing but still few studies have focused on the influence of microgravity on sleep, and the underlying mechanisms are not well understood; therefore, effective countermeasures are not yet available^6,8,12,17^.

Because of the specificity of space flight missions and the scarcity of space resources, it is difficult to conduct in-orbit studies, let alone the more systematic and in-depth studies on the influences and mechanisms^2,21–23^. Ground-based facilities for simulated microgravity have become important auxiliary research methods, such as head-down tilted bed rest for human^24,25^, tail suspension^26^ or hind limb unloading for rats^27^, random positioning machine (RPM) for plants, cells, fruit flies and so on^22,28–34^, etc., which have improved basic scientific research and the preparation for space experiments^23,35^. However, among these researches, the effects of simulated microgravity on sleep were rarely reported due to the limitations of experimental conditions, and most of them were the head-down tilted bed rest experiments, but volunteers were strictly limited on the bed which differed from the normal or in-orbit sleep condition. Among the various methods used to simulate the effects of microgravity, RPM has the characteristics of easy operation, continuous work, and stable and reliable performance and thus can be repeated in a short period of time, and is increasingly widely used^32,33,35,36^. In this study, the sample installation unit of RPM is designed with the corresponding sample platform with DAM2 to realize real-time behavior monitoring, and our previous study also suggested that it is feasible to use RPM to study the activity and sleep of fruit flies^36^.

*Drosophila melanogaster* is a convenient model for in-orbit research due to its small body size, fast reproduction, short life cycle, and relatively low life support requirements^23,37,38^. A number of studies have shown that fruit flies are sensitive to the space environment, which results in certain physiological and genetic responses^22,34,38–42^. In addition, fruit flies have been widely used in recent years as a model in sleep pattern research^43–46^. Some ground-based experiments showed that fruit flies could respond to simulated microgravity obtained through RPM^38^ and the gene expression observed in these experiments was partially similar to that observed in space flight experiments^22,23,34^. In addition, our previous study also suggested that the activity and sleep of fruit fly changed under RPM, and the increased activity was consistent with the relevant reports of current space experiments^2,36,42,47^.

On the basis of our previous study that adopted RPM for simulated microgravity to investigate the alterations of activity and sleep of fruit fly^36^, this study considered the roles of light conditions (normal photoperiod and total darkness), fruit fly strains (Canton-S and w1118), and sexes play in the effects of simulated microgravity on sleep of fruit fly, through real-time activity and sleep behavior monitoring and detection of relative expression levels of target genes, including major clock genes and some neurotransmitter-related genes. This study is expected to provide some new ideas and suggestions for the study of the effects of space microgravity on circadian rhythms or sleep of organisms.

## Results

### Activity and sleep rhythms under simulated microgravity

**Fig. 1:**
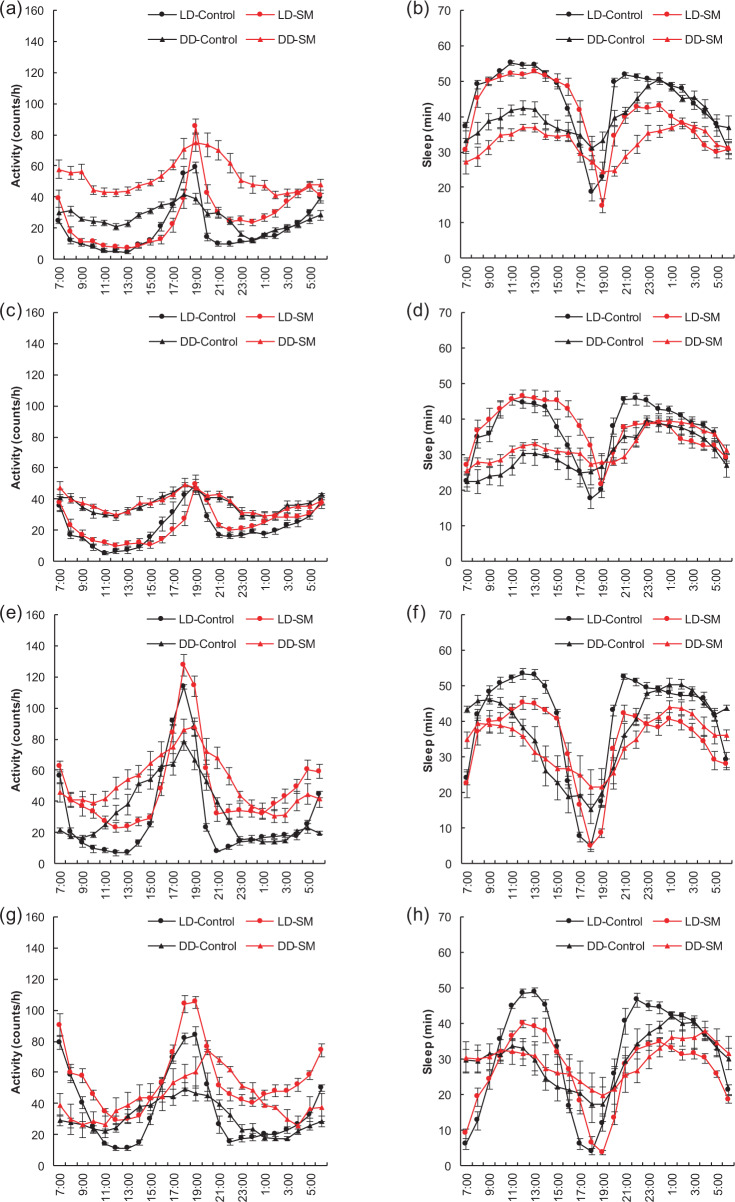
Activity and sleep rhythms under simulated microgravity under LD and DD conditions. **a** Activity rhythms of Canton-S males. **b** Sleep rhythms of Canton-S males. **c** Activity rhythms of Canton-S females. **d** Sleep rhythms of Canton-S females. **e** Activity rhythms of w1118 males. **f** Sleep rhythms of w1118 males. **g** Activity rhythms of w1118 females. **h** Sleep rhythms of w1118 females. Data were calculated by a mean of 3 days. *n* ≧ 90. Error bars represent standard error.

Under normal photoperiod (LD) and constant dark (DD) conditions, the data monitored continuously for 3 days under simulated microgravity were selected for a statistical analysis of activity and sleep rhythms (Fig. 1). Three-day average data showed that under LD condition, the simulated microgravity group (SM) exhibited similar activity and sleep rhythms as the control group (Control), regardless of Canton-S or w1118, male or female, with an activity peak and a sleep trough at ~6:00 and 18:00, the same as the set light/dark cycles (12 h/12 h, light-on time set at 6 am every day (GMT + 6:00))^48^. Except that, the amplitudes between the Control and SM groups were different. However, under DD condition, the activity and sleep rhythms of the Control and SM groups were disturbed to a certain extent, with Canton-S more obvious than w1118, but still maintaining a circadian rhythm.

### Activity and sleep patterns of Canton-S fruit fly

**Fig. 2:**
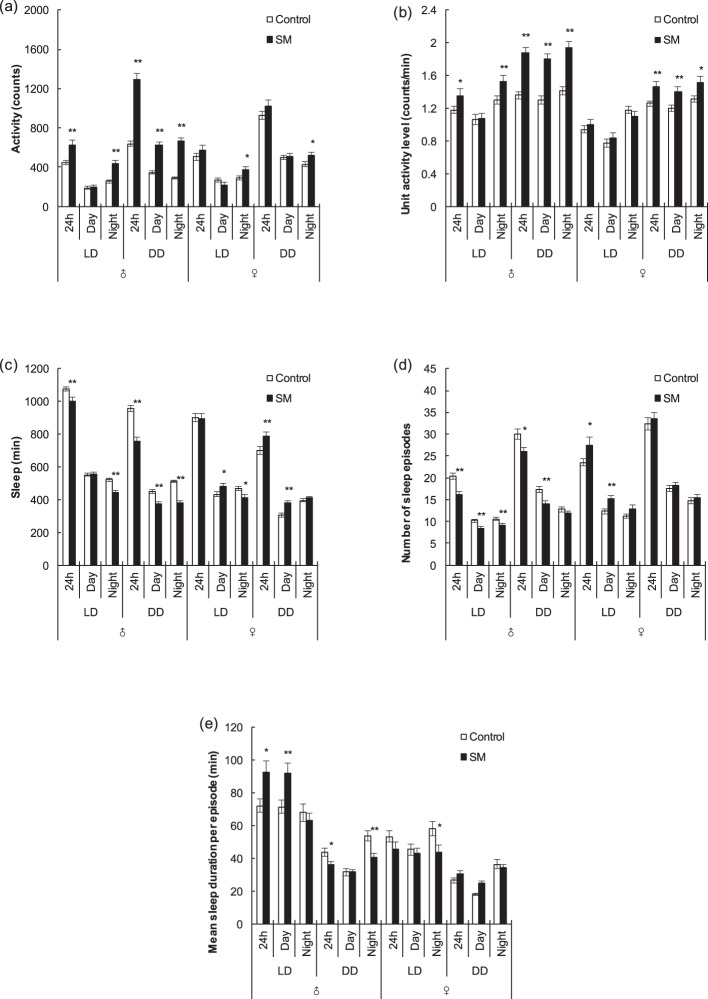
Activity and sleep of Canton-S male and female fruit flies under simulated microgravity under LD and DD conditions. **a** Total activity. **b** Unit activity level. **c** Total sleep time. **d** Number of sleep episodes. **e** Mean sleep duration per episode. Data were calculated by mean of 3 days. White and black boxes represent data for the control and simulated microgravity fruit flies, respectively. *n* ⫺ 90. *P < 0.05, **P < 0.01. Error bars represent standard error.

The activity results of Canton-S fruit fly in 3 days (Fig. 2a, b) showed that for LD condition under simulated microgravity, males’ total activity increased (*P* < 0.01), which was mainly due to the increased total activity at night (*P* < 0.01), increased unit activity level (*P* < 0.01) and decreased sleep time, and thus the awake time for activity increased (*P* < 0.01). However, in females, no statistically significant change in unit activity level was observed, although the total activity at night increased (*P* < 0.05) due to awake time for activity increasing (*P* < 0.05). For the DD condition under simulated microgravity, in both males and females, the SM group was more active than the Control group during both day and night. In males, the unit activity level increased (*P* < 0.01) and awake time for activity increased (*P* < 0.01), which both led to an increase in total activity (*P* < 0.01), and in females, the unit activity level increased (*P* < 0.01 during the day and *P* < 0.05 at night, respectively), and activity increased at night (*P* < 0.05).

Further analysis of sleep patterns in 3 days (Fig. 2c–e) showed that for LD condition under simulated microgravity, males’ total sleep time decreased (*P* < 0.01), primarily at night (*P* < 0.01) because the number of sleep episodes decreased (*P* < 0.01), but during the day, statistically significant changes in sleep time were not observed between the SM and Control groups, although the number of sleep episodes decreased (*P* < 0.01) and the mean sleep duration per episode increased (*P* < 0.01), indicating that sleep quality might increase, which is presumably related to homeostatic rebound sleep^49–53^ due to a lack of sleep and a high unit activity level at night. However, in females, statistically significant changes in total sleep time were not observed. At night, sleep time decreased (*P* < 0.05) because the mean sleep duration per episode decreased (*P* < 0.05), but during the day, sleep time increased (*P* < 0.05) because the number of sleep episodes increased (*P* < 0.01). For DD condition under simulated microgravity, males’ total sleep time decreased during both day and night (*P* < 0.01), with the mean sleep duration per episode decreasing at night (*P* < 0.01) and the number of sleep episodes decreasing during the day (*P* < 0.01). However, in females, the total sleep time increased (*P* < 0.01), which was mainly due to the mean sleep duration per episode increasing during the day (*P* < 0.01).

In summary, for LD condition under simulated microgravity, males’ activity increased and sleep decreased mainly at night, while in females, slight alterations in activity were observed, and sleep time decreased at night, which might result in homeostatic rebound sleep, making sleep time increase during the day. For DD condition under simulated microgravity, both males and females became more active. In males, sleep decreased during both day and night, which is more obvious than that under LD condition. However, in females, sleep time increased under simulated microgravity during the day, and long sleep increased, which might because it was not easy for fruit flies to wake up when lack of light, or additional sleep was needed to supplement consumption or the energy to remain awake because of the high unit activity level.

### Activity and sleep patterns of w1118 fruit fly

**Fig. 3:**
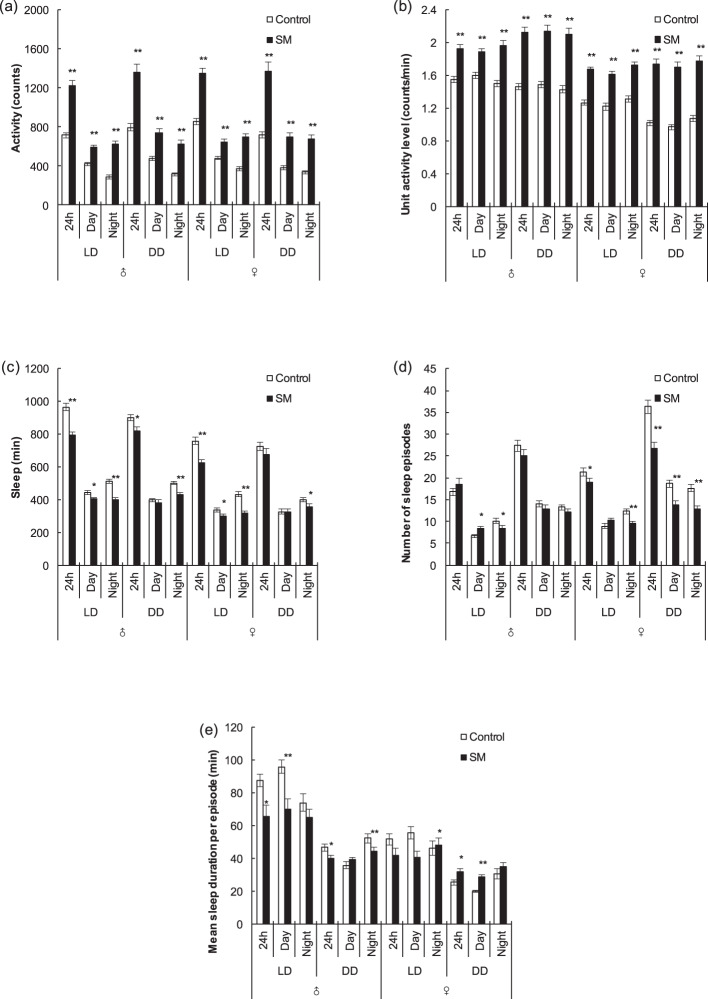
Activity and sleep of w1118 male and female fruit flies under simulated microgravity under LD and DD conditions. **a** Total activity. **b** Unit activity level. **c** Total sleep time. **d** Number of sleep episodes. **e** Mean sleep duration per episode. Data were calculated by mean of 3 days. White and black boxes represent data for the control and simulated microgravity fruit flies, respectively. *n* ≥ 90. *P < 0.05, **P < 0.01. Error bars represent standard error.

Further experiments were carried out using strain w1118. The activity results of w1118 fruit fly in 3 days (Fig. 3a, b) showed that under simulated microgravity, in both males and females, the SM group was more active than the Control group under both LD and DD conditions, with increasing unit activity level and total activity during both day and night (*P* < 0.01).

Further analysis of the sleep patterns (Fig. 3c–e) showed that for LD condition under simulated microgravity, in both males and females, sleep time decreased under simulated microgravity during both day and night (*P* < 0.05 during the day and *P* < 0.01 at night for males, all *P* < 0.01 for females, respectively). At night, the number of sleep episodes decreased (*P* < 0.05 for males, *P* < 0.01 for females, respectively), and during the day, the mean sleep duration per episode decreased (*P* < 0.01 for males, *P* < 0.05 for females, respectively), which meant sleep fragmentation and quality decreased. For DD condition under simulated microgravity, in both males and females, sleep time decreased mainly at night (*P* < 0.01, *P* < 0.05, respectively), and in males, the mean sleep duration per episode decreased (*P* < 0.05), and in females, the number of sleep episodes decreased (*P* < 0.01).

In summary, in terms of activity, both in males and females, the SM group was more active than the Control group under both LD and DD conditions, with increasing unit activity level and total activity. For sleep, both in males and females, the sleep time decreased at night under simulated microgravity under both LD and DD conditions. However, during the day, sleep also decreased under simulated microgravity under LD condition but not under DD condition, which we speculate might be because the fruit flies are awakened by light, or need more sleep to supplement their consumption or the energy to remain awake because of the hyperactivity under DD condition.

As indicated, the results of activity and sleep patterns of Canton-S and w1118 fruit fly showed that simulated microgravity had simple effects on activity and sleep, and these effects varied among in different light conditions (LD/DD), strains, and sexes. Therefore, we speculated that the effects of simulated microgravity might be affected and adjusted by these factors (strains, sexes, light conditions). MANOVA (multi-way analysis of variance) was performed, and the results showed that there were interactions of simulated microgravity effect and these factors. For unit activity level, in each strain and sex, the interaction between simulated microgravity and light conditions was found (*P* = 0.003), meaning that the effect of simulated microgravity was regulated by light conditions, which was more obvious in DD condition. And in LD condition, the interaction between simulated microgravity and strains was found (*P* = 0.003), meaning that the effect of simulated microgravity was different in each strain. For sleep time, in Canton-S males, the interaction between simulated microgravity and light conditions was found (*P* = 0.003). And in LD condition, the interaction between simulated microgravity and strains was found (*P* = 0.034). In addition, for number of sleep episodes, the interaction between simulated microgravity and sexes is found in both Canton-S and w1118 (*P* = 0.009, *P* = 0.004, respectively).

### Relative expression levels of target genes after simulated microgravity

In this study, the relative expression levels of major clock genes *per*, *tim*, *Clk*, *cyc* and *cry* of different strains and different sexes were detected after 3 days of simulated microgravity under LD and DD conditions.

**Fig. 4:**
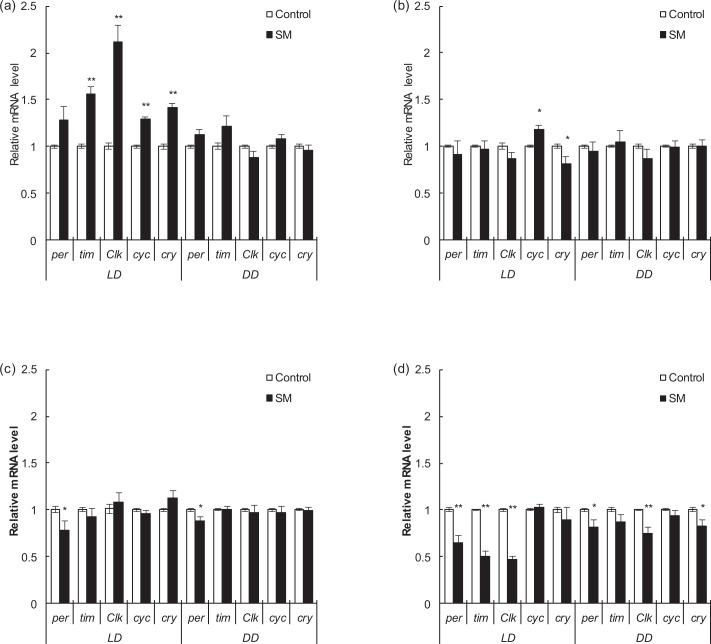
Relative expression levels of target major clock genes after 3 days of simulated microgravity under LD and DD conditions. **a** Canton-S males. **b** Canton-S females. **c** w1118 males. **d** w1118 females. White and black boxes represent data for the control and simulated microgravity fruit flies, respectively. *P < 0.05, **P < 0.01. Error bars represent standard error.

For Canton-S fruit flies, alterations were observed only under LD condition. After simulated microgravity, *tim*, *Clk*, *cyc,* and *cry* increased (*P* < 0.01) in Canton-S males (Fig. 4a), while *cyc* increased (*P* < 0.05) but *cry* decreased (*P* < 0.05) in Canton-S females (Fig. 4b). For w1118 fruit flies, after simulated microgravity, *per* decreased (*P* < 0.05) under both LD and DD conditions in both w1118 males and females (Fig. 4c, d). In addition, *tim* and *Clk* decreased under LD condition (*P* < 0.01) and *Clk* and *cry* decreased under DD condition (*P* < 0.05, *P* < 0.01, and *P* < 0.05, respectively) in w1118 females (Fig. 4d).

The relative expression levels of the *Ddc*, *ple*, *Trh,* and *Gad1* genes, which are related to the synthesis of the neurotransmitters DA, 5-HT, and GABA, were also detected as major clock genes above. Among them, DA promotes and maintains arousal, GABA promotes sleep, and 5-HT promotes arousal or sleep according to different receptor subtypes^53–56^.

**Fig. 5:**
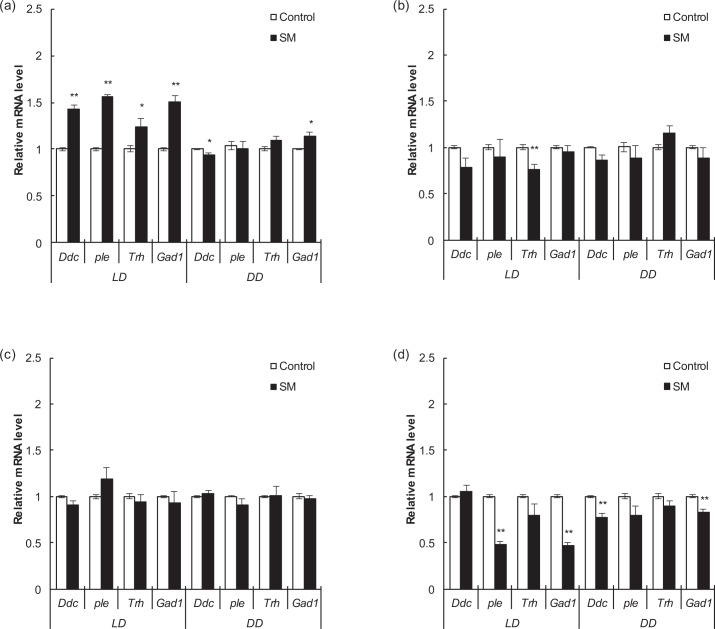
Relative expression levels of target neurotransmitter-related genes after 3 days of simulated microgravity under LD and DD conditions. **a** Canton-S males. **b** Canton-S females. **c** w1118 males. **d** w1118 females. White and black boxes represent data for the control and simulated microgravity fruit flies, respectively. *P < 0.05, **P < 0.01. Error bars represent standard error.

In Canton-S males, after simulated microgravity, the relative expression levels of *Ddc*, *ple*, *Trh,* and *Gad1* all increased under LD condition (*P* < 0.01, *P* < 0.01, *P* < 0.05, and *P* < 0.01, respectively) while *Ddc* decreased (*P* < 0.05) and *Gad1* increased (*P* < 0.05) under DD condition (Fig. 5a). Combined with the above results of activity and sleep, under LD condition, increased *ple* might promote the synthesis of DA, thus inhibiting sleep, and increased *Gad1* under both LD and DD conditions might be related to a feedback mechanism^53,57^ due to sleep deprivation and hyperactivity. However, in Canton-S females, only *Trh* decreased under LD condition (*P* < 0.01) (Fig. 5b). In w1118 males, after simulated microgravity, no statistically significant change was observed, either under LD or DD condition (Fig. 5c). However, in w1118 females, *ple* and *Gad1* decreased under LD condition (*P* < 0.01), and *Ddc* and *Gad1* decreased under DD condition (*P* < 0.01); among these, *Gad1* decreased under both LD and DD conditions, indicating that the synthesis of GABA might be affected, thus inhibiting sleep (Fig. 5d).

## Discussion

With the deepening of human space exploration, there is growing concern about astronauts’ health and space sickness. Researchers are still making great efforts to ensure that astronauts return safely and healthily; however, the disordered circadian rhythm and sleep require further research. In addition, with the continuous development of space life science, the demand for ground-based facilities for simulated microgravity is also increasing. RPM has two independent rotation mechanisms, and both can rotate at different and random speeds and directions to provide continuous random changes in orientation relative to the gravity vector and prevent the biological system from perceiving and responding to the gravitational acceleration vector^23,28,29^. RPM is increasingly widely used in ground-based experiments on microgravity effects on not only plants but also various organisms^23,32,33,35,36^. In our previous study^36^, we proposed to use RMP to study the effects of simulated microgravity on the activity and sleep of fruit fly, and developed the corresponding equipment, and demonstrated the feasibility of this strategy from many aspects such as behavior monitoring, growth, and development, oxidative stress, etc. We found that during simulated microgravity, fruit flies moved easily and freely, without swinging or flipping, and activity increased, which was consistent with the results of space flight^2,42,47^ and ground-based experiments^58,59^. Therefore, we thought it was feasible to study the effects of simulated microgravity on activity and sleep of fruit flies by RPM, and valuable references could be provided for aerospace medical research.

The circadian rhythm of the 24-h day is fundamental to life on Earth^12^, but astronauts are reported to suffer the circadian rhythm and sleep disorders during orbit, which might cause impaired alertness, concentration loss, diminished performance, and other dangerous problems that jeopardize productivity, health, and safety^6,7,9,13^. In our previous study^36^ mentioned above, we used Canton-S males under LD condition only, to provide the feasibility of RPM, and found that Canton-S males were more sensitive to short-time simulated microgravity, and the alterations of activity and sleep were different between day and night, suggesting the light might play an important role. In addition, some studies have also shown that the influence of microgravity on motility varies among different strains, ages, and sexes^23,37,60^, but the reasons remain speculative. Therefore, we think that to reveal the effects of simulated microgravity on activity and sleep of fruit fly might also need to carefully consider the influences of other key elements such as light conditions, strains, and sexes. On that basis, this study used relevant strategy, combined with the different light conditions (LD/DD), to systematically study the response of different strains and sexes fruit fly to the short-time simulated microgravity. For activity and sleep rhythms, they were all consistent with the photoperiod treatments regardless of the strain and sex under LD condition, although the amplitudes changed, as some studies showed^7^. However, under DD condition, all rhythms were disordered, especially under simulated microgravity, although in this study, the treatment time was so short that the fruit flies still retained part of their inherent circadian rhythm^53^. We speculate that as light is a strong zeitgeber, the effect of simulated microgravity might be too weak to be masked^41^. In addition, alterations in activity level and sleep patterns were also studied. In general, under LD condition, activity increased and sleep decreased under simulated microgravity, which occurred mainly at night in Canton-S but at both day and night in w1118, and these findings are similar to the results of our previous study on Canton-S males mentioned above^36^. The increased activity of fruit flies was observed in many space flight^2,42,47^ and ground-based experiments through parabolic flights^58^ and magnetic field^59^, and the abovementioned studies^9^ also showed that astronauts’ sleep decreased during orbit, which was consistent with the results in this study. There was also a study that showed that Canton-S males maintained normal locomotor activity rhythm and sleep pattern after spaceflight, but their test was started 48 h after landing, which might be enough for fruit flies to readapt to the Earth’s environment^41^. And in our study, we also found that after the exposure stopped, the fruit flies quickly returned to normal rhythms within 24 h. For the DD condition, all the fruit flies exhibited hyperactivity under simulated microgravity, which we speculate might lead to energy consumption increases, muscle reductions, and even loss of life, although it still needs further verification. In addition, except for Canton-S males, the fruit flies exhibited more sleep during the day than under LD condition, which we speculate might be due to the fruit flies being more drowsy and less likely to wake up because of lack of light or hyperactivity.

Some studies showed that during orbit, astronauts under a disordered photoperiod suffered more disordered circadian rhythms and sleep than under a normal photoperiod, and the changes caused by space flight were more obvious than under a normal photoperiod, including fatigue, posture balance ability, skeletal muscle strength and endurance, memory, reaction time, etc., and further increased the riskiness of space missions, and astronauts took medications more frequently, including sleep- and wake-promoting drugs and other medications^8^. In addition, some studies showed that adequate and appropriate light condition during the day, which is the basis of the normal circadian system, was conducive to keep astronauts awake and alert^12^. Thus, light systems are being developed to enhance the illumination of the working and living environment of astronauts and improve their sleep, circadian entrainment, and daytime alertness^15,61,62^.

The sleep-wake state of fruit flies is closely related to the circadian clock and neurotransmitters, which are highly conserved among species^63–66^. In our previous study on Canton-S males under LD condition, we also found that the major clock genes and some neurotransmitter-related genes were sensitive to simulated microgravity^36^. Therefore, we chose more related genes to further study the possible mechanisms based on the light conditions, strains, and sexes. In general, simulated microgravity led to changes in the major clock genes and neurotransmitter-related genes in all fruit flies. However, only the Canton-S males under LD condition and w1118 females under both LD and DD conditions responded positively to simulated microgravity, and other alterations were limited. There was also a study showed that the alterations of some clock genes were slight, but the fruit flies were collected 50 h after space flight, so they might readapt to the Earth’s environment^41^. Except that, researchers also found regulated output genes of the circadian clock system were affected, which could be further studied in the future. In addition, there is a bimodal activity pattern with a morning peak around lights-on and an evening peak before lights-off in fruit fly^53,67^, so we chose 18:00 when the light turned off as the sampling time, but as the clock genes exhibit their own circadian rhythmic patterns, the sampling time might affect the expression analysis results; therefore, analysis at multiple sampling time points spread across 24 h could be carried out in the future^41^. For neurotransmitters, previous results during space flight and ground-based experiments were often inconsistent and difficult to explain, mainly due to the complex and extreme space environment, the various types of experimental animals, the different experimental conditions, etc^4,68^.. For instance, some studies showed that no statistically significant change occurred in the contents of 5-HT in rats after simulated microgravity^69^, which was consistent with our previous experiment^36^, although other studies showed increased^70^ or decreased^57,71^ contents, and the results of DA^57,69,71^, GABA^57,71^ and other neurotransmitters were also inconsistent between different studies. Moreover, the feedback and homeostatic response should also be considered. In addition, the regulation relies not only on a single neurotransmitter but also on the whole nervous system^53,54^.

Although the alteration tendencies of behavior were similar, there were still many differences in alterations under simulated microgravity among strains and sexes, so as the alterations in major clock genes and neurotransmitter-related genes. Some studies have shown that the white-eyed fruit fly strain w1118 have enhanced light sensitivity but deficient visual acuity, contrast, and brightness, a substantial decrease in the number of synaptic vesicles of photoreceptor terminals^72^, and suffer from retinal degeneration including disorganization of the regular array of ommatidia, atrophied rhabdomeres, progressive loss of photosensitive units, and the abnormal electroretinogram^73,74^. As for the sexes, fruit flies have sexually dimorphic phenotypes and regulatory pathways in sleep^75^. For example, some genes affecting the rhythms of fruit fly were located on the X chromosome such as *per*^76^, *dusky*^77^, and *disconnected*^78^, which may lead to sexual differences due to dose effect^67^; the activity of dorsal clock neuron differs between males and females^79^; juvenile hormone and its receptor generate the sexual dimorphism of sleep through the sex differentiation-related genes regulated and independent of the circadian clock^75^.

In conclusion, this study showed that the light conditions (LD/DD), strains, and sexes would all altered the simulated microgravity effects on fruit flies’ activity and sleep individually or in combination (Supplementary Fig. 1). We speculate that the normal photoperiod could ease the effects of simulated microgravity and the appropriate photoperiod and lighting system with proper intensity, spectrum, and distribution might be powerful countermeasures for circadian and sleep disorders during orbit and should be prudent tested^8,15^. Besides, the differences in alterations under simulated microgravity among strains and sexes might provide some ideas for further research on the underlying mechanisms. Therefore, we speculate that unidentified pathways involved in the regulatory mechanism require further exploration, and we will conduct experiments with different strains (mutants), sexes, and light conditions, combined with the genetic methods and multi-omics methods to define the influence pathways of microgravity on sleep more clearly, and carry out in-depth research in the future.

In addition, it must be noted that although RPM is helpful for the study of space life, it cannot provide real microgravity^23,29,35^. Therefore, we are also actively looking for opportunities to carry out in-orbit experiments to verify the results and accurately explore the similarities and differences and restrictions on ground-based experiments^35,80^.

## Methods

### Fruit fly line and experimental conditions

Wild-type Canton-S and mutant w1118 fruit flies (Core Facility of Drosophila Resource and Technology, CEMCS, CAS) were used. Fruit flies were maintained on standard medium containing inactivated yeast, sucrose, agar, corn meal, and maltose in an artificial climate incubator with 25 ± 1 °C, 60 ± 2% relative humidity, and 12 h/12 h light/dark cycles (LD, light-on time set at 6 am every day (GMT + 6:00))^48^.

### Simulated microgravity

**Fig. 6:**
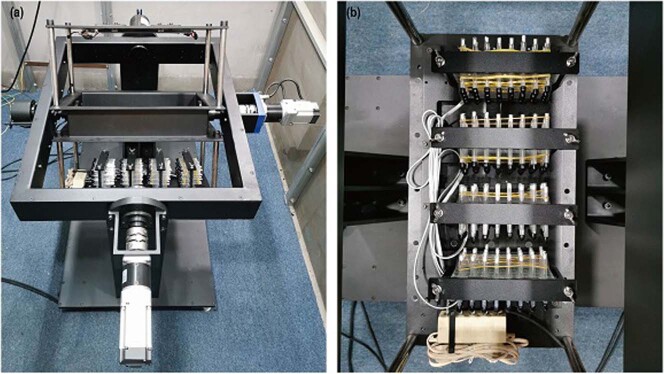
Microgravity simulation system. **a** Random positioning machine. **b** Sample installation unit.

The microgravity simulation system used in this study is jointly developed by the Institute of Urban Environment and Shanghai Institute of Technical Physics, Chinese Academy of Sciences, according to the characteristics of fruit fly, composed of the RPM and control system, which was described in our previous study^36^. The RPM mainly consists of a frame, a vertical support frame, an external rotation mechanism, an internal rotation mechanism, a drive system, a sample installation unit, and a light source (Fig. 6a). With random mode setting in this study, the rotation direction of the individual rotation mechanism was randomly varied, and the rotation velocity was randomly varied between 2–6 rpm, and the residual g level was about 10^−4^ g. Based on the characteristics of the Drosophila Activity Monitoring System (DAM2, Trikinetics, USA), the sample installation unit (Fig. 6b) is designed with the corresponding sample platform, to realize real-time transmission of monitoring data^36^. To solve the problem of light changes on the sample platform during the rotation of RPM, besides controlling the external illumination in laboratory, we also installed the LED light source in the RPM, which can control the illumination time and intensity stably and accurately. We detected the light intensity of the control group (laboratory light) and simulated microgravity group (SM group), which were 98 ± 2 lx and 110 ± 2 lx, respectively. According to our previous experiments, the change of light intensity of about 10 lx between the two groups would not have a significant impact on the activity and sleep of fruit fly.

### Behavior monitoring

One- to three-day-old male and female fruit flies were used for the experiments and divided into a control group (Control) and a simulated microgravity group (SM). According to the different strains and sexes, Control-Canton-S♂, Control-Canton-S♀, Control-w1118♂, Control-w1118♀, SM-Canton-S♂, SM-Canton-S♀, SM-w1118♂, and SM-w1118♀ were set up, and according to the light conditions, the LD group (normal photoperiod, 12 h/12 h light/dark cycles) and DD group (constant dark) were set up (Supplementary Fig. 2). The fruit flies were anesthetized with carbon dioxide.

Fruit flies were first loaded into DAM tubes (inner diameter 3.5 mm, length 65 mm) containing culture medium (5% sucrose/2% agar). The DAM2 with SM group was placed in the RPM and the Control group was placed next to the RPM before the RPM turned on for more than 24 h to make the fruit flies adapt the separately living environment as well as the light conditions, temperature, and humidity. Then, the RPM was turned on at 6 am for 3 consecutive days and then turned off. The differences in the total activity (activity counts), unit activity level (total activity counts/awake time), total sleep time, number of sleep episodes, and mean sleep duration per episode (total sleep time/number of sleep episodes) among the exposure groups were analyzed. The activity of each fruit fly was recorded every 5 min by a computer, and fruit fly was defined to be in sleep state when the time of immobility was longer than 5 min^41,81^. Each experiment was repeated three times.

### Detection of relative expression levels of target genes

Ten fruit flies in each group were collected into each EP tube after 3 consecutive days of treatment, quick-frozen with liquid nitrogen, and stored at −80 °C for follow-up detection. As the bimodal activity pattern with a morning peak around lights-on and an evening peak before lights-off in fruit fly^53,67^, samples were taken at 18:00 in this study. The relative expression levels of major clock genes (*period* (*per*), *timeless* (*tim*), *Clock* (*Clk*), *cycle* (*cyc*) and *cryptochrome* (*cry*)) and genes related to the synthesis of the neurotransmitters dopamine (DA), serotonin (5-HT) and gamma-aminobutyric (GABA) (*dopa decarboxylase* (*Ddc*), *pale* (*ple*), *tryptophan hydroxylase* (*Trh*), and *glutamic acid decarboxylase 1* (*Gad1*)) were detected. Total RNA was extracted by TRIzol reagent and reverse transcribed by the PrimeScript RT Master Mix Perfect Real Time kit (Takara, Japan), and the Green Premix Ex TaqII kit (Takara, Japan) was used for real-time fluorescence quantitative PCR experiments. All samples were tested three times, and the CT values of the target genes were normalized to the CT values of the reference gene rp49^82^. The relative quantitative analysis was carried out by the 2^−ΔΔCT^ method^83^. The primers used are shown in Supplementary Table 1. The experiment was repeated three times.

### Statistical analysis

SPSS 25 and Microsoft Excel 2016 were used for the statistical analysis. The differences between the Control and SM groups in each strain, sex and light condition were analyzed by one-way ANOVA and Student’s *t* test. MANOVA (multi-way analysis of variance) was performed to study the interactions of simulated microgravity effect and factors (strains, sexes, light conditions). The statistical results are expressed as the mean ± SEM.

### Reporting summary

Further information on research design is available in the Nature Research Reporting Summary linked to this article.

## Supplementary information

Supplementary information

Reporting Summary

## Data Availability

The authors declare that the data supporting the findings of this study are available within the paper.
