# Supplementary material for: Alterations in the activity and sleep of Drosophila melanogaster under simulated microgravity
Source: NPJ Microgravity. 2021 Jul 22;7:27. doi: 10.1038/s41526-021-00157-5 (PMC8298474; doi:10.1038/s41526-021-00157-5)
Supplement: Supplementary file 1 — Supplementary information [file 41526_2021_157_MOESM1_ESM.pdf]

Supplementary information

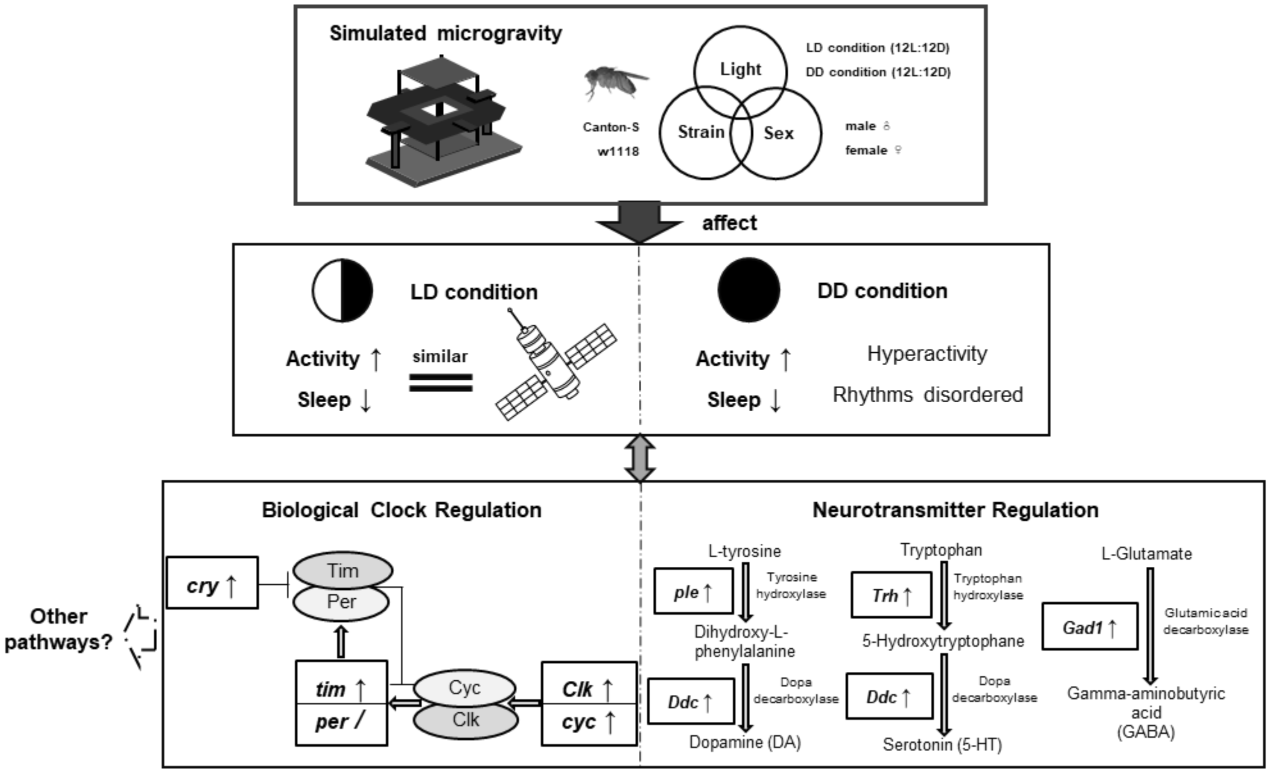

Supplementary Fig. 1 Summary of this study. Simulated microgravity was obtained through the random positioning machine, and the arrows indicate the alterations in the corresponding parameters, with "↑" indicating an increase and "↓" indicating a decrease. Because the sensitivity of each gene to simulated microgravity differs among the LD/DD conditions, strains, and sexes, the relative expression levels of target genes of Canton-S males under LD conditions are shown as examples.

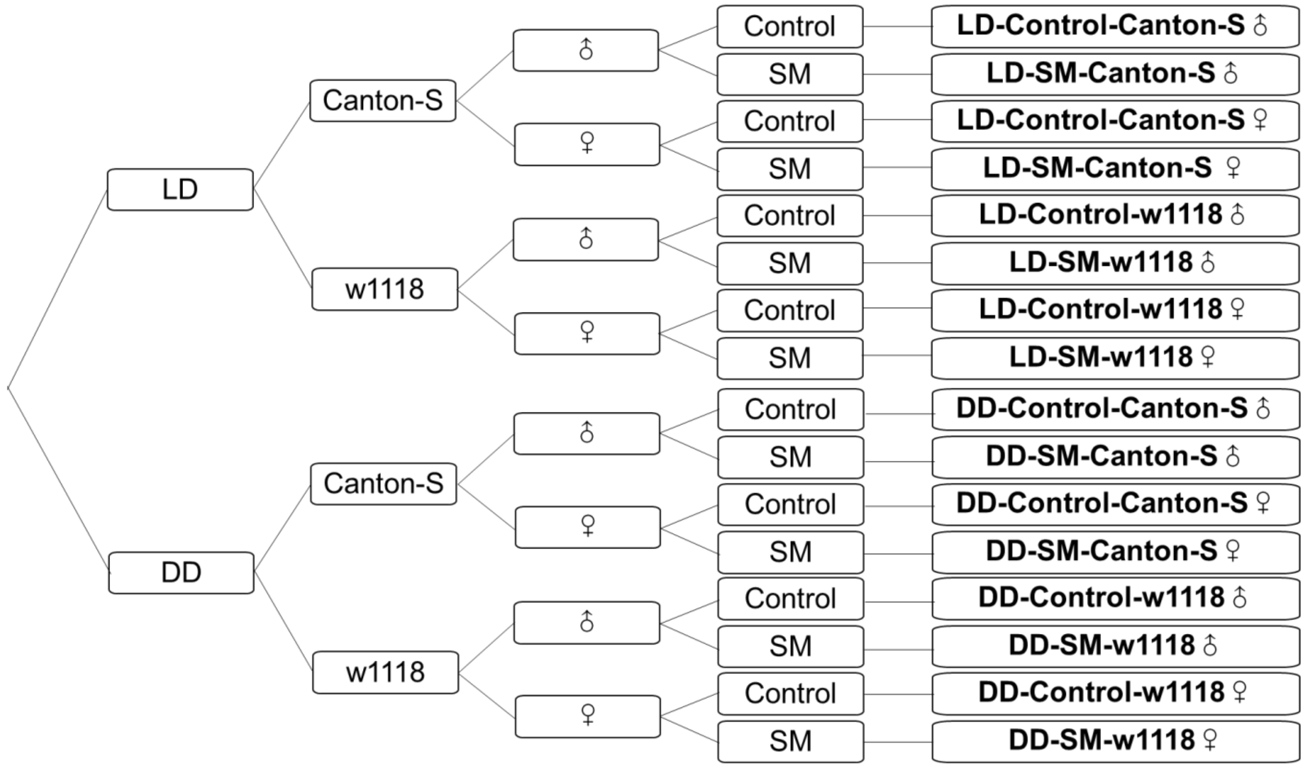

Supplementary Fig. 2 Groups design. LD and DD: light conditions (normal photoperiod (LD) and constant dark (DD)); Canton-S and w1118: strains; ♂ and ♀: sexes; SM: simulated microgravity group; Control: control group.

Supplementary Table 1. Primer sequences

Gene	Forward primer sequences (5'-3')	Reverse primer sequences (3'-5')
per	ACACCATCGCGTCTTTCAGG	ATTCGCGTGTTCCGGCTCTG
tim	CAACACTACCAACCCAACGTCC	GAGCGTGTGCCGAGGTGG
Clk	ACGAGAGCGACGACAAGGATG	GGCTATCGTGGACTTCAGGACC
cyc	GCGAAAGCTGGACAAACTGACT	CCGTTGAATGGATGTAAGCTGC
cry	CGATGGAGAGAGTGCAGGTA	GTCCATCAGTTGCCGCCT
Ddc	CAATCCACACAAATGGATGCT	AAGAGGGTCCACATTGAACGC
ple	AATGTCAAGGCTCCGTGGTTC	AATCCGGGGTGGTTCATGTC
Trh	CTTCGGCGATATGGTGTGGT	TTGAATCCTGGATGGTCGGC
Gad1	ATGTCGCTGAATCCCAACGG	TCAGCAGGAACTCCCGTGTC
rp49	AGCATACAGGCCCAAGATCG	GTTGTCGATACCCTTGGGCT
